# A systematic review on the accuracy of manufacturing techniques for cobalt chromium fixed dental prostheses

**DOI:** 10.1080/26415275.2020.1714445

**Published:** 2020-01-20

**Authors:** Per Svanborg, Lars Hjalmarsson

**Affiliations:** a Department of Prosthodontics/Dental Materials Science, Institute of Odontology, Sahlgrenska Academy, University of Gothenburg, Göteborg, Sweden; b Specialist Dental Clinic, Folktandvården Sörmland AB, Mälar Hospital, Eskilstuna, Sweden; c Centre for Clinical Research Sörmland, Uppsala University, Eskilstuna, Sweden

**Keywords:** Cobalt chromium, fixed dental prosthesis, accuracy, fit, manufacturing technique

## Abstract

**Purpose:**

To compare the fit and assess the accuracy of tooth-supported single and multi-unit FDPs in cobalt chromium fabricated using different manufacturing techniques.

**Materials and methods:**

A systematic search was performed in three databases; PubMed, Scopus, and Web of Science, using clearly specified search terms and inclusion criteria. The search yielded 1071 articles and included 18 articles in the analysis. Data regarding the fit analyses and the methods of manufacturing were extracted and the accuracy was defined as the fit result minus the pre-set cement spacer. Internal gap (IntG) was the mean of all the internal measuring points and total gap (TotG) was the mean of all measuring points (marginal, cervical, chamfer, axial, occlusal).

**Results:**

The total gap results for fit and accuracy irrespective of manufacturing technique were 96 μm and 54 μm for single crowns, 107 μm and 54 μm for multi-unit FDPs, and 98 μm and 54 μm for both single crowns and multi-unit FDPs combined. For total gap of single crowns soft milling had the highest accuracy, for multi-unit FDPs additive manufactured restorations had the highest accuracy. With the results grouped by impression technique, the accuracy for total gap was highest for digital impressions and lower for conventional impressions.

**Conclusions:**

Due to the inherent limitations of this systematic review, it still remains unclear what effect the manufacturing technique has on the fit of FDPs. However, the descriptive results suggest that the marginal fit of cobalt chromium FDPs is not negatively affected by the manufacturing technique.

## Introduction

The manufacturing of dental restorations in cobalt-chromium alloys (CoCr) can be done using different production techniques. The traditional technique is the lost wax technique, a formative technique also called casting, but the lost wax technique is being rapidly replaced by computer-aided design/computer-aided manufacturing (CAD/CAM) techniques in dentistry and dental technology. Today, CAD/CAM techniques dominate restoration manufacturing in dentistry. For the production of CoCr, these techniques include both subtractive and additive manufacturing (AM), such as, computer numeric controlled (CNC) milling and laser melting, and the quality and fit of these restorations need to be evaluated.

An internal or marginal misfit can affect the cement junction and lead to dissolution, which may cause loosening of the restoration or secondary caries [1]. The fit of a restoration is influenced by several factors, including the preparation type and taper, the amount of cement used, the pressure during cementation and the viscosity of the cement [2–4]. Studies on marginal gap have debated on what distances are to be considered as clinically acceptable. Most authors agree on a mean marginal gap of approximately 100 μm [1,5–8]. For an internal gap, 120 μm has been reported as acceptable [1,7].

When assessing the internal fit of a restoration, or the cement film thickness, the fit is measured as the discrepancy at the chamfer or cervical area gap, axial discrepancy and occlusal discrepancy. It can also be presented as a mean of all the measuring areas/points [9,10]. To measure the fit, several techniques are used which are either destructive or nondestructive. With the destructive technique, the restoration is cemented onto dies or extracted teeth and then embedded into e.g. epoxy resin, and sectioned for analysis using a microscope [11]. Nondestructive techniques can be the direct view technique, examination using an explorer with a specified radius, the silicone or impression replica technique, micro-computed tomography (micro CT), and the optical three-dimensional (3D) scanning technique [7,12–16].

Studies on the fit of restorations report the distance between the tooth and restoration and use both precision and accuracy interchangeably as the term for fit. However, the accuracy of a manufacturing technique is the closeness of the produced physical object to the virtual object or a master object and the precision of a manufacturing technique is the closeness of the results of repeated manufacturing. Accuracy has been defined as the deviation from the original object and precision as the accuracy of repeated measurements [17]. Precision has also been defined as the ability of a machine tool or manufacturing process to produce a component to close tolerances [18]. In regard to these definitions, accuracy can be seen as the closeness of the fit of a dental restoration to the aimed fit, and precision as a measurement of the reliability of a machine or a manufacturing technique. So, in order to measure the accuracy of a tooth-supported fixed dental prosthesis (FDPs), the settings for the marginal and internal cement spacer must be provided and used to compare with the fit results. Nevertheless, discussions regarding the cement spacer settings are uncommon in the literature and several studies on precision and accuracy of tooth-supported FDPs fail to provide the settings in the manuscript or compares groups with different spacer settings without taking the spacer into consideration when drawing conclusions.

This systematic review will focus on studies evaluating the fit of tooth-supported fixed prostheses in CoCr produced with different manufacturing techniques, with clearly defined spacer settings of the aimed cement space.

### Aim

To compare the fit and assess the accuracy of tooth-supported single and multi-unit FDPs in CoCr fabricated using different production techniques.

## Material and methods

The present study followed the PRISMA statement for transparent reporting of systematic reviews and meta-analyses [19].

### Search strategy

The electronic searches were performed on 31 Oct 2017 in three databases: PubMed, Scopus and Web of Science, and limited to English, Swedish, Danish and Norwegian languages. The searches and terms were:

### Search pubmed 2017-10-31

((((((FDP[Title/Abstract] OR fixed partial denture[Title/Abstract] OR FPD[Title/Abstract]))) OR (((prosthesis[Title/Abstract] OR prostheses[Title/Abstract])) AND ((Dental OR dentistry)))) OR ((crown[Title/Abstract] OR crowns[Title/Abstract] OR bridge[Title/Abstract] OR bridges[Title/Abstract])))) AND ((cobalt chromium[Title/Abstract] OR cobalt chrome[Title/Abstract] OR CoCr[Title/Abstract] OR ‘Co Cr’[Title/Abstract] OR CrCo[Title/Abstract] OR ‘Cr Co’[Title/Abstract] OR ‘Cr-Co’[Title/Abstract] OR ‘Co-Cr’[Title/Abstract] OR cobalt-chromium[Title/Abstract] OR chromium-cobalt[Title/Abstract] OR chrome-cobalt[Title/Abstract] OR cobalt-chrome [Title/Abstract]))

### Search pubmed 2017-10-31 using mesh terms

((‘Dental Prosthesis’[Mesh]) AND ‘Chromium Alloys’[Mesh]) AND (gap OR gaps OR fit OR misfit OR precision OR accuracy)

### Search scopus and web of science 2017-20-31

#### Search 1

cobalt chromium OR cobalt chrome OR CoCr OR ‘Co Cr’ OR CrCo OR ‘Cr Co’

OR ‘Cr-Co’ OR ‘Co-Cr’ OR cobalt-chromium OR chromium-cobalt OR chrome-cobalt OR cobalt-chrome

TITLE-ABS-KEY (prosthesis OR prostheses AND (dental OR dentistry)) OR TITLE-ABS-KEY (crown OR crowns OR bridge OR bridges OR fdp OR fixed AND partial AND denture OR fpd) AND TITLE-ABS-KEY (cobalt AND chromium OR cobalt AND chrome OR cocr OR ‘Co Cr’ OR crco OR ‘Cr Co’ OR ‘Cr-Co’ OR ‘Co-Cr’ OR cobalt-chromium OR chromium-cobalt OR chrome-cobalt OR cobalt-chrome)) AND (LIMIT-TO (LANGUAGE, ‘English’))

#### Search 2

FDP OR fixed partial denture OR FPD OR prosthesis OR prostheses AND (Dental OR dentistry) OR crown OR crowns OR bridge OR bridges

TITLE-ABS-KEY (prosthesis OR prostheses AND (dental OR dentistry)) OR TITLE-ABS-KEY (crown OR crowns OR bridge OR bridges OR fdp OR fixed AND partial AND denture OR fpd) AND TITLE-ABS-KEY (cobalt AND chromium OR cobalt AND chrome OR cocr OR ‘Co Cr’ OR crco OR ‘Cr Co’ OR ‘Cr-Co’ OR ‘Co-Cr’ OR cobalt-chromium OR chromium-cobalt OR chrome-cobalt OR cobalt-chrome)) AND (LIMIT-TO (LANGUAGE, ‘English’))

### Inclusion and exclusion criteria

The inclusion criteria comprised studies of tooth-supported prostheses in CoCr with the following information described; fit assessment, measurement techniques, manufacturing technique (lost-wax, hard and soft CNC-milling, additive manufacturing), and pre-set cement spacer. Studies not meeting all inclusion criteria, studies of implant-supported prostheses and studies measuring fit after ceramic veneering were excluded.

### Study selection

The authors independently screened the titles and abstracts from the studies found in the electronic searches described above, considering the inclusion criteria. Any disagreements were resolved after discussion. After selection, the full texts of the studies were acquired. These publications were again independently screened, and a final discussion took place to reach a consensus.

### Extraction of data

Data from the included studies were independently extracted and registered in data extraction forms by the authors. The extracted data was checked and disagreements between the authors were discussed until a consensus was reached. In this review, the marginal gap (MG) was defined as the shortest distance from the preparation line to the inside of the restoration or from the most cervical edge of the restoration to the tooth [20]. The internal gap (IntG) was the mean of all the internal measuring points (cervical, chamfer, axial, occlusal) and the total gap (TotG) was the mean of all the measuring points available in the studies (marginal, cervical, chamfer, axial, occlusal).

From the included studies, the following data was extracted (when available): Year of publication, Preparation type, Master model material, Impression technique, Restoration (crown or multi-unit FDP), Number of test specimens or patients, Pre-set spacer, CAD software, CAM, Manufacturing technique (Formative, Subtractive, Additive), Alloy, Fit analysis technique, Measuring points, Analysis area (absolute marginal gap, marginal gap, cervical area gap, axial gap, occlusal gap, internal gap, total gap).

### Analyses

In the present review, mean values were calculated as weighted values based on the individual group mean value and number of test specimens or patients per group. Fit was the mean of distances reported in the studies and accuracy was the fit minus the pre-set spacer. The analyses were done for single crown and multi-unit FDPs, separately and combined. The SPSS (IBM SPSS v.24.0) statistics software was used for statistical analysis.

## Results

### Studies

**Figure 1.**
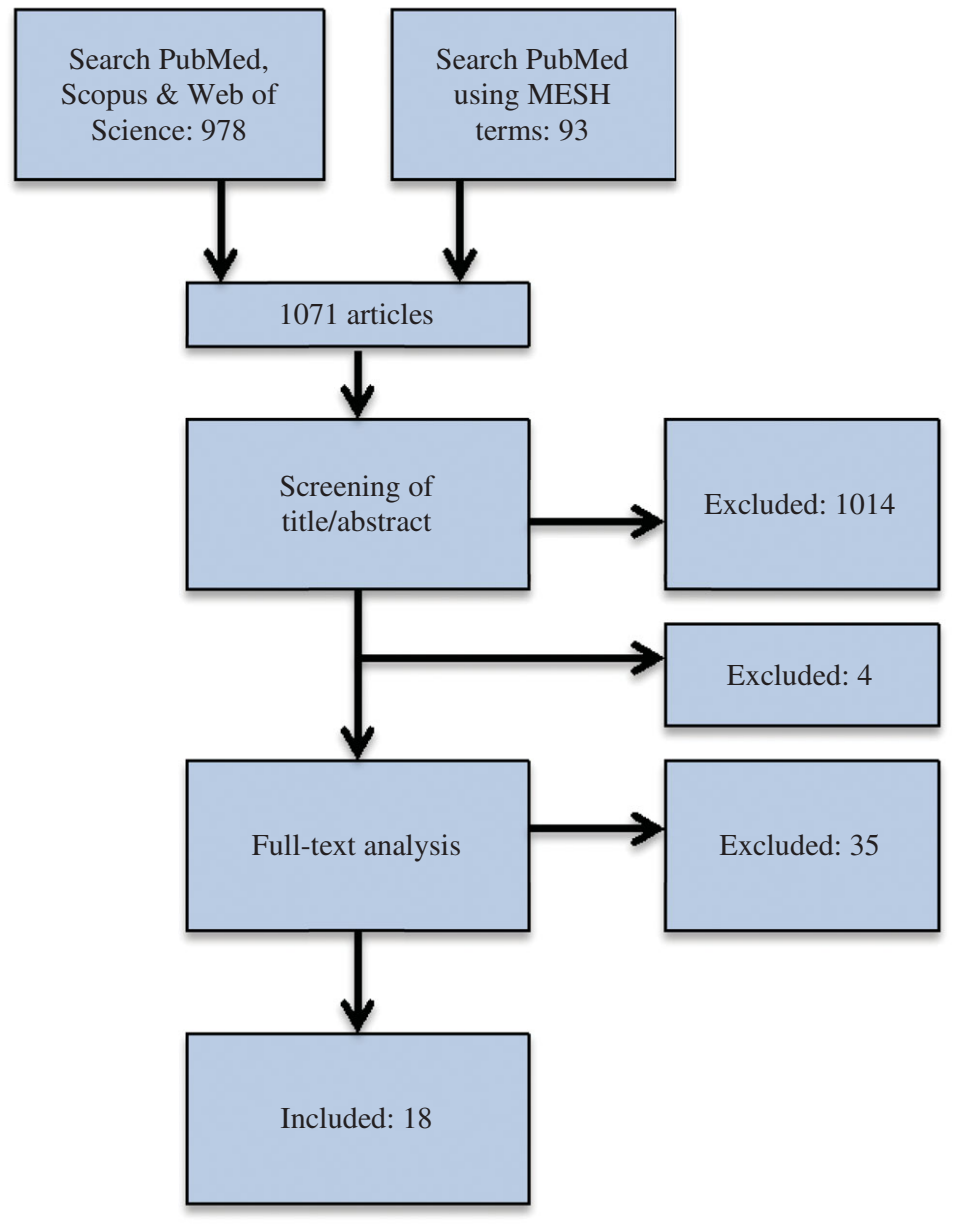
Search strategy.

The first searches done in PubMed, Scopus and Web of Science, resulted in 978 articles. In addition, a new PubMed search was done using mesh terms. After removal of duplicates, another 93 articles were added. The authors screened the titles and abstracts from the 1071 studies found independently, considering the inclusion criteria. After discussion, disagreements were resolved resulting in 57 articles which were analyzed in full-text by the authors independently of each other. A final discussion took place with the aim to reach a consensus, and another four articles were excluded, one due to wrong material (NiCr) [21] and three since they were only conference reports [22–24]. Hence, 53 articles were left for the final full-text analysis. With the inclusion and exclusion criteria applied, 35 articles were excluded. As a result, 18 articles met the inclusion criteria (See Figure 1 and Table 1).

**Table 1.** The included studies and the results they present regarding impression technique, tooth area, manufacturing technique, pre-set cement spacer, number of test specimens and reported gaps (in micrometer).

	Settings					Marginal gap			Internal gap			Total gap		
Author	Impression	Tooth	Man Tech^a^	Pre-set spacer	*N*	Fit	SD^b^	Acc^c^	Fit	SD	Acc	Fit	SD	Acc
*Single crowns*														
Ates et al. [36]	Conv^d^	PM^f^	CLW^j^	0 (0.5)–30	10	25	4	25						
	Conv	PM	HM^k^	0 (0.5)–30	10	52	11	52						
	Conv	PM	AM^l^	0 (0.5)–30	10	28	6	28						
Dahl et al. [37]	Conv	INC^g^	LW^m^	0–20	3				64	30	44	58	23	38
	Dig^e^	INC	HM	50–90	3				162	153	72	90	78	0
	Dig	INC	AM	55–80	3				106	53	26	82	37	2
Gunsoy et al. [38]	Conv	PM	LW	50	16	86	20	86	107	27	57	97		47
	Dig	PM	CLW	0 (0.5)–50	16	85	19	85	93	21	43	92		42
	Dig	PM	HM	0 (0.5)–50	16	84	18	84	90	20	40	89		39
	Dig	PM	AM	0 (0.5)–50	16	52	11	52	82	17	32	66		16
	Conv	M^h^	LW	50	16	96	19	96	149	23	99	121		71
	Dig	M	CLW	0 (0.5)–50	16	90	17	90	118	21	68	104		54
	Dig	M	HM	0 (0.5)–50	16	88	16	88	117	21	67	103		53
	Dig	M	AM	0 (0.5)–50	16	40	10	40	83	10	32	58		8
Harish et al. [39]	Conv	PM	CLW	20	10	177	26	157	187	11	167			
	Conv	PM	AM	20	10	102	17	82	108	11	88			
Huang et al. [40]	Conv	M	LW	70	110	91	36	21	196	87	126	161	70	91
	Conv	M	AM	70	110	76	33	6	219	76	149	170	81	101
Kim et al. [41]	Conv	M	CLW	30	10	124	52	94	170	56	140	159	55	129
Kim et al. [42]	Conv	M	LW	0 (0.5)–30	10							64	14	34
	Conv	M	SMill^n^	0 (0.5)–30	10							33	5	3
	Conv	M	AM	0 (0.5)–30	10							47	9	17
Kocaagaoglu et al. [43]	Conv	M	CLW	0 (0.5)–30	10	102	26	102	131	23	101	121	24	91
	Conv	M	SMill	0 (0.5)–30	10	72	28	72	159	23	129	130	24	100
	Conv	M	Mill	0 (0.5)–30	10	68	12	68	143	26	113	118	21	88
	Conv	M	AM	0 (0.5)–30	10	73	15	73	149	27	119	124	23	94
Lovgren et al. [44]	Conv	M	CLW	0 (0.5)–50	10	104	33	104	134	24	84	127	27	77
	Conv	M	HM	0 (0.5)–50	10	91	24	91	126	22	76	117	22	67
	Conv	M	AM	0 (0.5)–50	10	53	19	53	111	19	61	96	19	46
Park et al. [45]	Conv	CA^i^	LW	0 (1)–25	10	34	8	34	69	21	44	53	33	28
	Conv	CA	SMill	0 (1)–25	10	62	16	62	109	22	84	88	39	63
	Conv	CA	HM	0 (1)–25	10	37	8	37	75	9	50	56	31	31
Park et al. [46]	Conv	CA	LW	0 (1)–25	10	40	10	40	77	10	52	58	31	33
	Conv	CA	HM	0 (1)–25	10	63	18	63	110	23	85	89	39	64
	Conv	CA	AM	0 (1)–25	10	71	19	71	128	23	103	103	43	78
Sundar et al. [13]	Conv	PM	AM	0 (0.5)–30	10	56	11	56						
*FDPs*														
Keul et al. [47]	Conv	4-unit	HM	30 (0.5)–60	12	91	91	61	143	75	83	130	79	70
	Dig	4-unit	HM	30 (0.5)–60	12	57	27	27	116	45	56	101	41	41
Kim et al. [48]	Conv	3-unit	AM	0 (0.5)–30	10	113	50	113						
Nesse et al. [49]	Conv	3-unit	LW	20	10				116		96			
	Conv	3-unit	HM	0 (0.5)–50	10				95		45			
	Conv	3-unit	AM	0 (0.5)–50	10				156		106			
Svanborg et al. [50]	Conv	3-unit	HM	30 (0.5)–60	10							117	12	54
	Dig	3-unit	HM	30 (0.5)–60	10							93	8	33
Ueda et al. [51]	Conv	4-unit	HM	30 (1.5)–60	12	81	66	53	98	62	38	94	63	34
	Dig	4-unit	HM	30 (1.5)–60	12	32	35	2	60	30	0	53	31	7
Örtorp et al. [52]	Conv	3-unit	LW	0 (0.5)–50	8	81	40	81				133	86	83
	Conv	3-unit	CLW	0 (0.5)–50	8	112	46	112				118	79	68
	Conv	3-unit	HM	0 (0.5)–50	8	152	91	152				166	136	116
	Conv	3-unit	AM	0 (0.5)–50	8	49	35	49				84	58	34

^a^Manufacturing technique; ^b^Standard deviation; ^c^Accuracy; ^d^Conventional; ^e^Digital; ^f^Premolar; ^g^Incisor; ^h^Molar; ^i^Canine; ^j^CAD/lost-wax; ^k^Hard milling; ^l^Additive manufacturing; ^m^Lost wax; ^n^Soft milling.

### Description of included studies and analyses

All results included in this review were of CoCr restorations before ceramic veneering. One study was clinical and involved patients and one used human teeth as master models. Twelve of the studies included single crowns and six measured the fit of multi-unit FDPs. Since most of the studies presented results from more than one test group the review resulted in 36 test groups with single crowns, four test groups with 4-unit FDPs and ten test groups with 3-unit FDPs. Most of the groups were comprised of ten specimens or patients but ranged from three to 110. Thirteen studies presented results from conventional impression techniques only and five studies compared conventional and digital impression techniques. The abutment teeth used in the studies were incisors, canines, premolars, and molars. The manufacturing techniques used were; eight test groups by CAD design with subtractive or additively produced wax patterns for the lost wax technique (CLW), nine test groups by the lost wax technique (LW), three test groups by soft milling (SMill), 16 test groups by hard milling (HM), and 14 test groups by additive manufacturing (AM). Several different CAD/CAM systems and alloys were used in the studies (Table 2).

**Table 2.** The different machines, CAD/CAM-systems and alloys presented in the included studies.

Casting machine	CAD system	CAM system	Alloy
Mikrotek N/S^a^ [36]	3Shape D700 [38,42,44,48]	Yenadent N/S [36]	BEGO Wirobond 280 [37,44]
BEGO Fornax [39,45,46]	3Shape D800 [45,46]	Yenadent DC40 [43]	BEGO Wirobond C [40,48,49,52]
BEGO Nautilus CC [44,52]	3Shape D640 [52]	Concept laser M1 [36,38,42,44]	BEGO N/S [36]
Renfert N/S [40]	3Shape N/S [37]	Eosint M270 [13,39,43,46,48]	EOS MP1 [39]
N/S [37,38,41,48,49]	3M Lava [51]	BEGO N/S [40]	EOS SP2 [13,42,43,46,48]
	Straumann Cares [47,50]	Straumann milling [47,50]	Straumann Coron [47,50]
	Dental Wings 3-series [36,43]	Ceramill Motion2 [43,45,48]	Degudent StarloyC [41,45,46]
	BEGO N/S [40]	Roeders RXD5 [44]	Ceramill Sintron [43,45,48]
	Identica Blue/ Exocad [41]	Datron D5 [45,46]	Gialloy CB [44]
	Ceramill Mind [48]	3M Lava CNC 500 [51]	Remanium Star CL [44]
	Ceramill Match2 [43]	Wieland Zeno 4820 [52]	Kulzer Cara milled [49]
	EOS/ Cambridge [43]	Biomain N/S [52]	Kulzer Cara SLM [49]
		Dentware N/S [37]	ACF LunaNEM [52]
		3D Systems Projet1200 [41]	Whitepeaks Coprabond K [36]
			Dentaurum N/S [36]
			Eisenbacher ED Kera-Disc [37,43,45,46]
			Eisenbacher ED Kera-C [43]
			Dentware CoCr [37]
			N/S [51,52]

^a^ N/S: Not specified.

**Figure 2.**
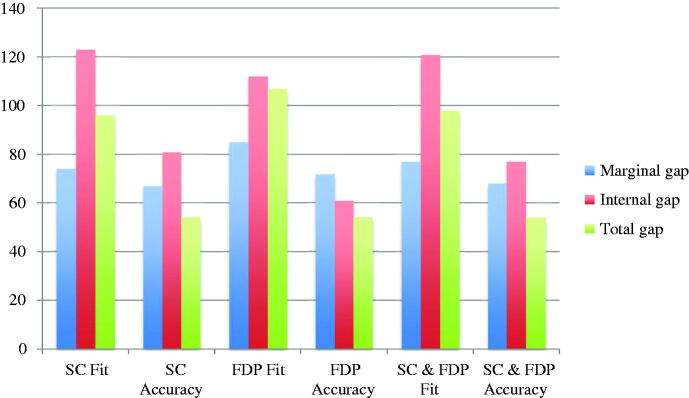
Fit and accuracy (μm) of single crowns (SC), multi-unit fixed dental prostheses (FDP) and combined divided by marginal gap, internal gap and total gap.

**Figure 3.**
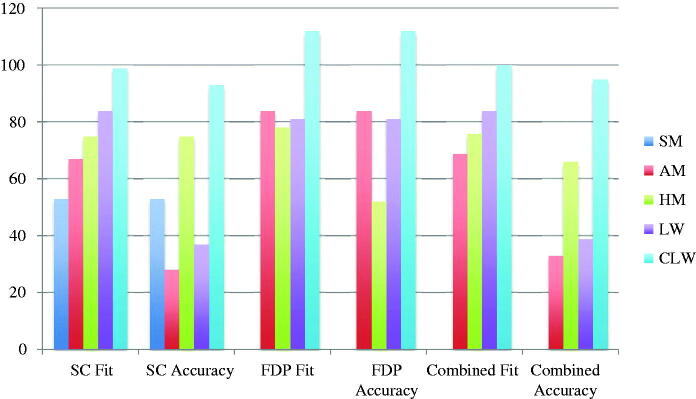
Marginal gap fit and accuracy (μm) of single crowns (SC), multi-unit fixed dental prostheses (FDP) and combined divided by production technique. SM: Soft milling, AM: Additive manufacturing, HM: Hard milling, LW: Lost wax, CLW: CAD lost wax.

**Figure 4.**
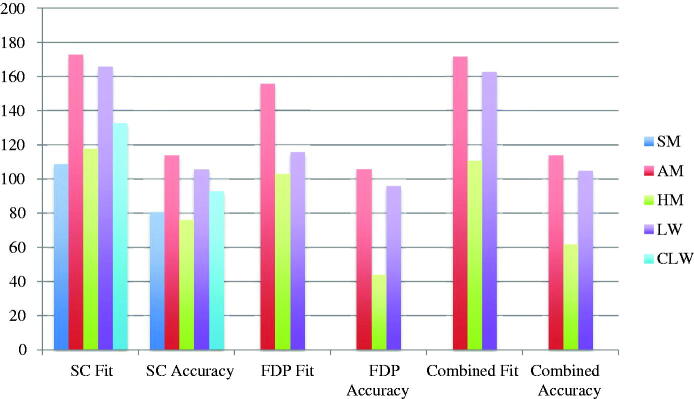
Internal gap fit and accuracy (μm) of single crowns (SC), multi-unit fixed dental prostheses (FDP) and combined divided by production technique. SM: Soft milling, AM: Additive manufacturing, HM: Hard milling, LW: Lost wax, CLW: CAD lost wax.

**Figure 5.**
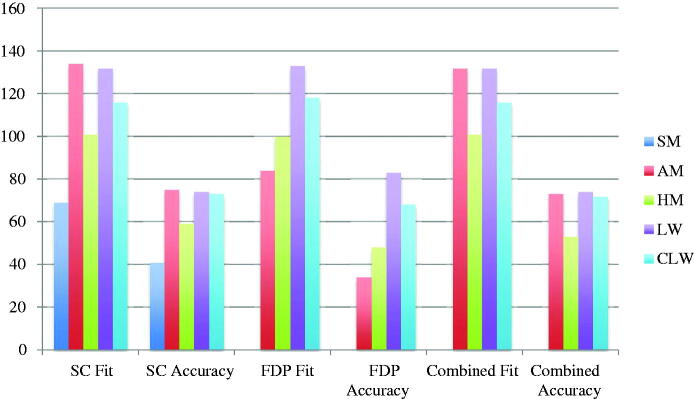
Total gap fit and accuracy (μm) of single crowns (SC), multi-unit fixed dental prostheses (FDP) and combined divided by production technique. SM: Soft milling, AM: Additive manufacturing, HM: Hard milling, LW: Lost wax, CLW: CAD lost wax.

**Figure 6.**
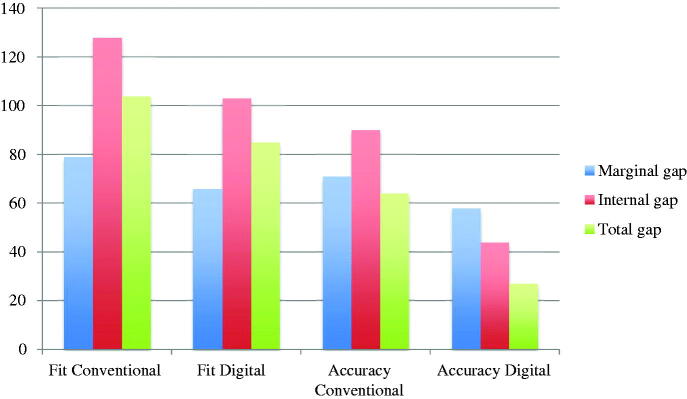
Fit and accuracy (μm) of conventional and digital impressions divided by marginal gap, internal gap and total gap.

The included studies reported results from fit measurements, the results are presented as means for single crowns, multi-unit FDPs and combined (single crowns and multi-unit FDPs). Also, the results are given as fit (the distance between model/tooth and restoration) and accuracy (fit minus the pre-set spacer). The fit and accuracy results for total gap irrespective of manufacturing technique were 96 μm and 54 μm for single crowns, 107 μm and 54 μm for multi-unit FDPs, and 98 μm and 54 μm for both single crowns and multi-unit FDPs combined (Figure 2). For manufacturing technique and the measuring area MG, AM single crown restorations presented the highest accuracy and CLW the lowest accuracy. For multi-unit FDPs in MG, HM presented the highest accuracy and CLW the lowest. The combination of single crowns and multi-unit FDPs showed AM as the technique with the highest accuracy and CLW as the one with the lowest accuracy (Figure 3). For internal gap the manufacturing technique with the highest accuracy for single crowns was HM followed by SMill. AM had the lowest accuracy. Internal gap for multi-unit FDPs showed that HM had the highest accuracy and AM the lowest. CLW and SMill did not present any results for multi-unit FDPs (Figure 4). For total gap and accuracy of single crowns SMill had the highest accuracy, for multi-unit FDPs AM had the highest (Figure 5). With the results grouped by impression technique, the accuracy for total gap was highest for digital impressions and lower for conventional impressions (Figure 6).

## Discussion

The aim of this systematic review was to compare the fit and assess the accuracy of tooth-supported single and multi-unit FDPs in CoCr manufactured by different techniques. Thus, the ambition of the authors was to compare the mean fit and mean accuracy values from the different manufacturing techniques. Although the number of included studies (*n* = 18) was relatively high, considering the strict inclusion and exclusion criteria, the diversity among the studies makes it hard to draw many reliable conclusions. The presented manufacturing techniques included four casting machines, 12 CAD systems and 14 CAM systems. There were both clinical and laboratory studies. In all the 18 included studies, conventional impression techniques were used, and in five of them digital impression techniques were also applied. In addition, the included studies examined both single crowns (*n* = 12) and multi-unit FDPs (*n* = 6), 19 different CoCr-alloys were used, and the fit measurements were performed with three different techniques. These variations lead to a shortage of sufficient data for each single variable of interest for further analyses. Therefore, the results from the present systematic review are mainly descriptive, and the results should be regarded as indicating trends.

After full-text analysis, 35 studies were excluded. Among these, 14 were excluded due to no mention of pre-set cement spacer. Measurements that take place without a clearly defined cement spacer only reflect the total deviation from the master model and not the actual accuracy. As a consequence, regardless of whether or not these non-mentions have been done intentionally, the results regarding fit from the 14 studies are most difficult to interpret and far from the clinical reality.

The comparison of studies on fit is a complex undertaking, not least since so many different parameters can affect the fit of a tooth-supported FDP. The present review focused on manufacturing technique, type of impression and measuring technique. As a consequence, a limitation of the present review is that other parameters, that also potentially could have affected the results presented in the included studies, have not been analyzed. Among these are cementation pressure, preparation type, type of master model, and model material. Also, the included studies do not mention internal adjustments before fit analyses, which could have affected the results. Restorations produced using the lost-wax technique have most likely been adjusted since the process of casting will create an oxide layer and there is always a risk of casting fins, rough surfaces, and gas porosities [25]. In a review by Nawafleh et al., the reliability of methods to measure marginal adaptation of crowns and FDPs was evaluated [26]. They concluded that the differences in testing methods and parameters between the studies result in a lack of consensus regarding marginal adaptation.

The commonly used manufacturing techniques (lost-wax, hard milling and additive manufacturing) showed no major differences in fit values for marginal gap: 84 µm, 76 µm, and 69 µm, respectively. The accuracy for the same techniques was 37 µm, 75 µm, and 28 µm respectively. The results from the present systematic review revealed that no single manufacturing technique was superior to the others regarding accuracy for both single crowns and multi-unit FDPs, nor for all different measuring areas (MG, IntG and TotG). The diversity in parameters makes it problematic to draw any conclusions regarding manufacturing technique. Interestingly, all techniques, except CLW multi-unit FDPs, presented a fit within 100 µm for MG and would, therefore, be considered clinically acceptable according to earlier publications (1, 5-8). For internal and total gap however, 120 µm have been considered clinically acceptable (1, 7), and therefore AM, LW and CLW could be regarded as clinically unacceptable in those areas. The accuracy of 3 D printed dental casting patterns has been shown to deviate from the designed dimensions, which could explain the CLW results [27]. Further, for the subtractive technique, different milling procedures have been shown to affect the accuracy [28]. In the present review, only marginal gap measurements are presented to represent the fit at the margin. According to Holmes et al., the absolute marginal gap (AMG) is a more relevant measurement since it captures any horizontal or vertical discrepancies [20]. It was the author’s ambition to use AMG. However, only three of the studies presented AMG measurements. The effect of the manufacturing technique on the fit of CoCr restorations has been evaluated regarding implant-supported restorations. For screw-retained full-arch FDPs, the manufacturing technique (HM and AM) did not significantly affect the fit [29]. For cement-retained FDPs of different span lengths, LW had the lowest marginal discrepancy for 5-unit FDPs, and AM had the smallest marginal discrepancy for 3 and 4-unit FDPs [30]. An earlier systematic review on the effect of manufacturing technique on marginal adaptation came to similar results; no clear conclusion could be drawn regarding the superiority of a specific technique [31].

When the studies were divided into conventional and digital impressions, the results indicate that the digital impression technique had a higher accuracy, especially in internal and total gap. This corroborates the results by Haddadi et al., who compared the accuracy of crowns based on digital oral scanning and conventional impressions using a split-mouth randomized study design [32]. In their study, crowns from digital impressions showed significantly better marginal and internal adaptation before cementation compared to conventional impressions. For full arch FDPs and longer span multi-unit FDPs, studies comparing digital and conventional impressions show no significant differences but suggest a more careful approach to using digital impression techniques [33–35].

Based on these descriptive results, the fit of FDPs should be evaluated using a detailed protocol disclosing all parameters that could affect the results. In a comparative study, all FDPs should be manufactured using the same settings and with the same post-processing strategies, if possible. For evaluation of the fit at the margin, the absolute marginal gap should be the measuring area used.

## Conclusions

Due to the inherent limitations of this systematic review, it still remains unclear what effect the manufacturing technique has on the fit of FDPs. However, the descriptive results suggest that the marginal fit of cobalt chromium FDPs is not negatively affected by the manufacturing technique.
